# Host Prdx6 contributing to the intracellular survival of *Brucella suis* S2 strain

**DOI:** 10.1186/s12917-019-2049-8

**Published:** 2019-08-22

**Authors:** Lu-Lu Wang, Xiao-Feng Chen, Pan Hu, Shi-Ying Lu, Bao-Quan Fu, Yan-Song Li, Fei-Fei Zhai, Dan-Di Ju, Shi-Jun Zhang, Yi-Ming Shui, Jiang Chang, Xiao-Long Ma, Bing Su, Yu Zhou, Zeng-Shan Liu, Hong-Lin Ren

**Affiliations:** 1 Key Laboratory of Zoonosis Research, Ministry of Education, Institute of Zoonosis / College of Veterinary Medicine, Jilin University, Xi An Da Lu 5333, Changchun, 130062 China; 2 State Key Laboratory of Veterinary Etiological Biology, Key Laboratory of Veterinary Public Health of the Ministry of Agriculture, Key Laboratory of Veterinary Parasitology of Gansu Province, Lanzhou Veterinary Research Institute, Chinese Academy of Agricultural Sciences, Lanzhou, 730046 China; 3 Shaheyan Animal Husbandry and Veterinary Medicine Station of Dunhua City, Dunhua, 133700 China

**Keywords:** Host, Prdx6, *Brucella*, Macrophage, Intracellular survival

## Abstract

**Background:**

Brucellosis is a worldwide zoonotic infectious disease that is transmitted in various ways and causes great harm to humans and animals. The brucellosis pathogen is *Brucella*, which mainly resides in macrophage cells and survives and replicates in host cells. However, the mechanisms underlying *Brucella* survival in macrophage cells have not been thoroughly elucidated to date. Peroxiredoxin 6 (Prdx6) is a bifunctional protein that shows not only GSH peroxidase activity but also phospholipase A2 activity and plays important roles in combating oxidative damage and regulating apoptosis.

**Results:**

Recombinant mouse (*Mus musculus*) Prdx6 (MmPrdx6) was expressed and purified, and monoclonal antibodies against MmPrdx6 were prepared. Using the *Brucella suis* S2 strain to infect RAW264.7 murine macrophages, the level of intracellular Prdx6 expression first decreased and later increased following infection. Overexpressing Prdx6 in macrophages resulted in an increase in *B. suis* S2 strain levels in RAW264.7 cells, while knocking down Prdx6 reduced the S2 levels in cells.

**Conclusions:**

Host Prdx6 can increase the intracellular survival of *B. suis* S2 strain and plays a role in *Brucella* infection.

## Background

The intracellular pathogen of *Brucella* spp. can cause brucellosis [1], which is one of the most important zoonoses worldwide, especially in developing countries, where epidemics may be severe. *Brucella* mainly parasitic in cells causes undulant fever [2], abortion and infertility in mammals, besides it leads to long-term severe complications in humans. Survival and replication properties of *Brucella* in host cells contribute to its virulence [3]. *Brucella* influencing antigen presentation mediated by cells can survive and replicate in professional and nonprofessional phagocytic cells [4]. It is reported that *Brucella* also take part in modulating events of signalling in host adaptive immune responses [3]. Furthermore, inhibiting phagosome-lysosome fusion has been regarded as a mechanism for *Brucella* intracellular survival [4]. However, the precise factors involved in those processes have not been clarified [4].

In our previous study, using different virulent *Brucella* strains to infect sheep (*Ovis aries*), we constructed a time-course suppression subtractive hybridisation (SSH) cDNA library of the buffy coat to analyse the transcriptional profiles of sheep infected by *Brucella*; in the SSH cDNA library, peroxiredoxin 6 (Prdx6) mRNA exhibited differential expression in buffy coats [2, 5, 6]. Peroxiredoxins is a Se-independent peroxidases superfamily and widely distributed in all phyla [7]. Prdx6, which shows GSH peroxidase (NSGPx) activity and phospholipase A2 (PLA2) activity [8] is the only 1-Cys member of the peroxiredoxin superfamily in mammalian [9]. The antioxidant activities of Prdx6 protect against oxidative stress [9, 10] and provide cell signalling [11]. Membrane damage associated with phospholipid peroxidation can be solved by over-expressing Prdx6 in cells [12], while lipid peroxidation and apoptosis occur if the expression of Prdx6 is blocked by an antisense oligonucleotide [13]. Prdx6 is also considered to help host to resist bacterial infection [14]. Furthermore, 1-Cys Prdx of bacteria can limit the oxidative damage to the pathogen to some extend and might therefore be one of bacterial survival-related factors [15].

In this study, according to our previous work, we focused on the relationship between Prdx6 and brucellosis. The open read frame (ORF) DNA sequence of mouse (*Mus musculus*) *Prdx6* (*MmPrdx6*) was cloned, and the recombinant MmPrdx6 (rMmPrdx6) protein with DNA protection activity was expressed. Thereafter, we prepared a monoclonal antibody (mAb) against MuPrdx6 for further detection of differential expression. By regulating the expression level of MmPrdx6 using overexpression and shRNA interference, intracellular bacterial levels in the RAW264.7 murine macrophage cell line infected with bacteria were detected to analyse the relationship between host Prdx6 and *B. suis* S2 strain infection. This study may contribute to the identification of critical molecular mechanisms in *Brucella* infection for better prevention and treatment of brucellosis.

## Results

### Expression and bioactivity analysis of MmPrdx6

The recombinant MmPrdx6 expression plasmid was constructed using the pET-28a vector with the inserted ORF of Prdx6, which had a length of 675 bp, encoding a protein of 224 amino acid residues, with a predicted molecular weight of 25.01 kDa. According to sequencing, the inserted ORF of *MmPrdx6* showed 100% sequence identity to a *Mus musculus Prdx6* gene (GenBank accession number AK168223.1). Positive clones were screened through bacterial PCR, and the integrity of the plasmids was verified by DNA sequencing, revealing 100% sequence identity to the *Prdx6* gene. The recombinant plasmids were used to express the rMmPrdx6 protein.

**Fig. 1:**
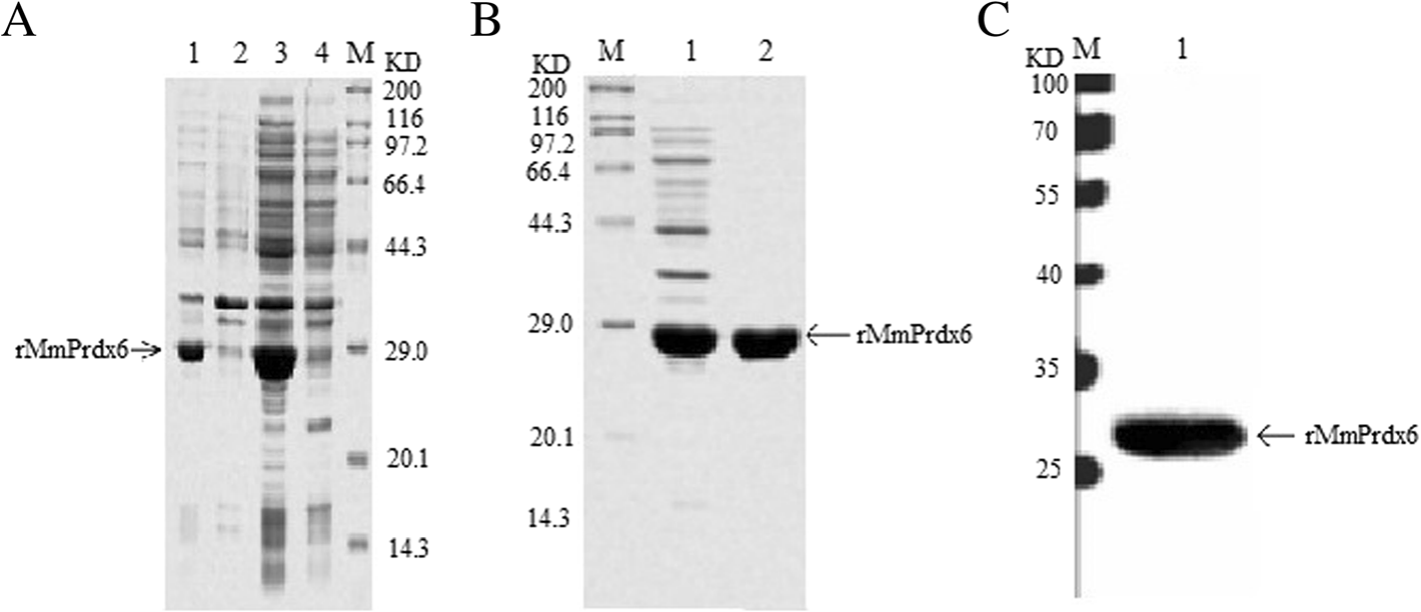
Expression and purification of rMmPrdx6. **a** Expression of the recombinant MmPrdx6 protein. M: Protein marker (Sangon, China); Lane 1: Total protein from induced *E. coli* BL21(DE3) cells harbouring pET-28a-Prdx6; Lane 2: Inclusion bodies of the induced *E. coli* BL21(DE3) cells harbouring pET-28a-Prdx6; Lane 3: Bacterial supernatants of the induced *E. coli* BL21(DE3) cells harbouring pET-28a-Prdx6; Lane 4: Total protein from uninduced *E. coli* BL21(DE3) cells harbouring pET-28a-Prdx6. **b** Purification of recombinant MmPrdx6 with a Ni-NTA column and visualization by SDS-PAGE. M: Protein marker; Lane 1: Bacterial supernatant of induced *E. coli* BL21(DE3) harbouring pET-28a-Prdx6; Lane 2: Purified rMmPrdx6 (with His-tag); **c** Identification of recombinant MmPrdx6 by western blotting. M: protein marker (Sangon, China); Lane 1: Purified rMmPrdx6

The positive clone harbouring the *pET-28a-Prdx6* plasmid was cultured in 5 mL of LB medium until the OD600 reached 0.4–0.6. Then, IPTG was added to the culture at a final concentration of approximately 1 mM, and the cells were subsequently incubated at 37 °C for 6 h to induce the expression of recombinant protein. The results of SDS-PAGE showed an approximately 28 kDa band in the induced cells, indicating that the recombinant Prdx6 protein was successfully expressed in the transformed *E. coli* BL21(DE3) cells compared to uninduced cells (Fig. 1a). As the 6 × His tag was fused to the N-terminus of the recombinant Prdx6 protein, an immobilized metal affinity chromatography method was utilized for rapid one-step purification employing an Ni-NTA column. The recombinant MmPrdx6 protein was purified and assessed via SDS-PAGE (Fig. 1b) and western blotting (Fig. 1c) using a commercial anti-His tag antibody (Sino Biological Inc.). The protein showed the expected molecular weight of approximately 28 kDa, including the molecular weight of the 6 × His-tag.

**Fig. 2:**
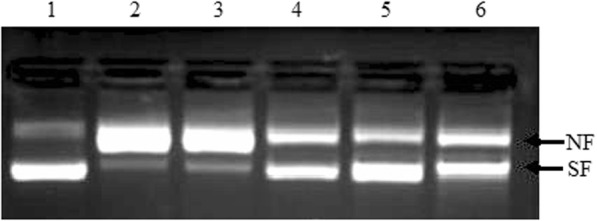
Analysis of the activity of recombinant MmPrdx6. Lane 1: Plasmid + (7 mM) phosphate buffer; Lane 2: Plasmid + MCO; Lane 3: Plasmid + MCO + BSA; Lane 4–6: Plasmid + MCO + rMmPrdx6 (0.08, 0.16, and 0.32 mg/ ml respectively); NF: Oxidized incomplete plasmids; SF: Supercoiled plasmids

The antioxidant activity of rMmPrdx6 was determined via the metal-catalysed oxidation (MCO) assay, in which the protein oxidized the plasmid in the supercoiled conformation. After the addition of rMmPrdx6, the amount of the oxidized incomplete plasmid and the degree of oxidation of the supercoiled plasmid were significantly decreased. Moreover, different concentrations of the rMmPrdx6 protein reduced the degree of oxidation of the plasmid, with 0.16 mg / mL rMmPrdx6 exhibiting the most obvious antioxidant activity (Fig. 2, lane 5). However, no protective ability preventing the supercoiled plasmid from being oxidized was found in the blank control group (Fig. 1d, lane 1) or the BSA control group (Fig. 1d, lane 3). Therefore, the antioxidant activity of the recombinant mouse Prdx6 protein was proven.

### Purification and specificity of the monoclonal antibody

**Fig. 3:**
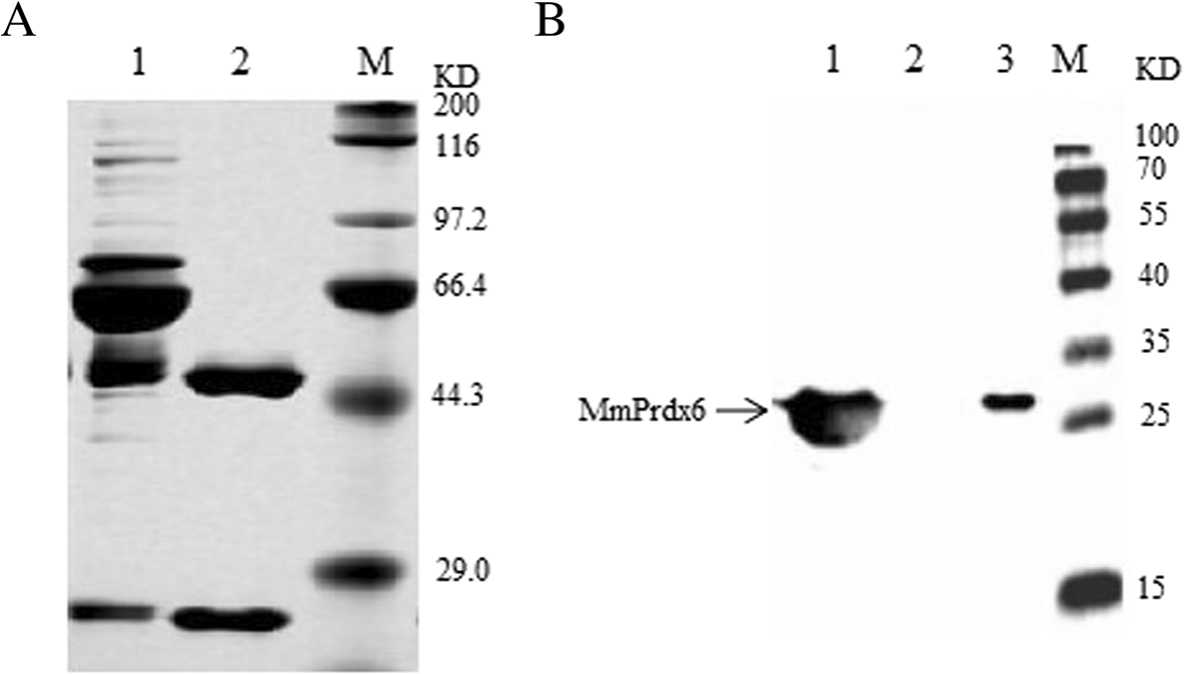
Purification and specificity analysis of the anti-MmPrdx6 monoclonal antibody. **a** mAbs against MmPrdx6 analysed via SDS-PAGE. M: Protein marker (Sangon, China); Lane 1: Ascites fluid harvested from BALB/c mice inoculated with the B5F2 hybridoma cell line secreting anti-MmPrdx6 mAbs; Lane 2: Purified mAbs against MmPrdx6. **b** Specificity of anti-MmPrdx6 mAbs determined through western blotting. M: Protein marker (Sangon, China); Lane 1: Purified rMmPrdx6 protein; Lane 2: Total protein from uninduced *E. coli* BL21(DE3) cells harbouring pET-28a-Prdx6; Lane 3: Total protein from the RAW264.7 murine macrophage cell line

A hybridoma cell line (B5F2) secreting mAbs against MmPrdx6 was obtained, and ascites fluid containing a large number of mAbs from B5F2 cells was purified to collected anti-MmPrdx6 mAbs with a high-performance liquid chromatography system (GE ÄKTA purifier100, Sweden). The purified mAbs with heavy chains (50 kDa) and light chains (25 kDa) were detected via SDS-PAGE (Fig. 3a). The subtype of the B5F2 monoclonal antibody was determined to be IgG3.

An immunological specificity assay was carried out using western blotting. Total protein including the native MmPrdx6 protein from RAW264.7 cells and the rMmPrdx6 protein was separated via 12% SDS-PAGE, followed by transfer to a PVDF membrane (Millipore, USA). Then, the PVDF membrane containing the protein samples was probed with the prepared anti-MmPrdx6 mAbs at a dilution of 1:5000, after which the appropriate secondary antibody conjugated with horseradish peroxidase-labelled goat anti-mouse IgG (dilution at 1:2000; BOSTER, China) was added to bind the primary anti-MmPrdx6 mAb. The results showed that the anti-MmPrdx6 mAbs were able to bind the rMmPrdx6 and native MmPrdx6 proteins extracted from RAW264.7 cells (Fig. 3b).

### Bacterial-induced differential expression of MmPrdx6 in RAW264.7 cells

**Fig. 4:**
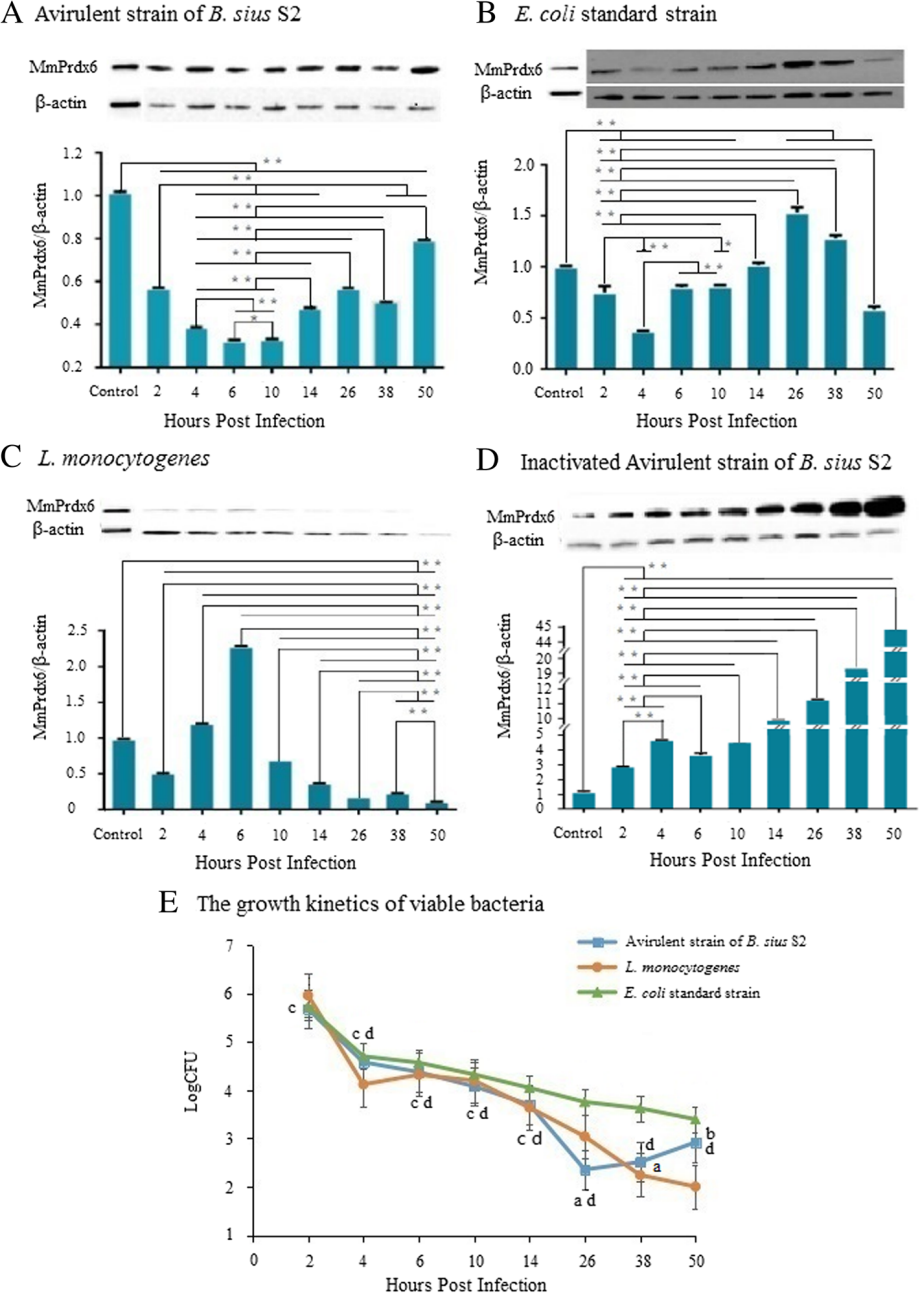
Prdx6 differential expression and counting of intracellular viable bacteria after bacterial infection. **a** Differential MmPrdx6 expression after infection by the avirulent strain of *B. suis* S2. **b** MmPrdx6 differential expression after infection by the *E. coli* standard strain. **c** MmPrdx6 differential expression after infection by *L. monocytogenes*. **d** Differential MmPrdx6 expression after challenge with the inactivated *B. suis* S2 strain. **e** Growth kinetics of intracellular viable bacteria at different times post-infection. Statistical analysis was conducted by one-way analysis of variance (ANOVA) using SPSS 13.0 software. * *P* < 0.05, ** *P* < 0.01. a: *p* < 0.05 vs. *E. coli*-infected group; b: *p* < 0.05 vs. *L. monocytogenes*-infected group; c: *p* < 0.05 vs. 50 h post infection in the S2-infected group; d: *p* < 0.05 vs. 2 h post infection in the S2-infected group

The expression profiles of MmPrdx6 in the RAW264.7 macrophage cell line infected with the avirulent S2 strain of *B. suis*, inactivated S2, *L. monocytogenes* and the *E. coli* standard strain were analysed using western blotting at different time points. Additionally, the total number of viable intracellular bacteria was counted. The expression levels of MmPrdx6 at all time points were lower in the S2-infected group than in the normal control group and were downregulated from 2 to 6 h post-infection, followed by upregulation from 10 to 50 h post-infection (Fig. 4a). In contrast to the S2-infected group, MmPrdx6 expression in the *E. coli*-infected group was downregulated from 2 to 4 h post-infection, then upregulated from 4 to 26 h post-infection, followed by downregulation after 26 h post-infection, and the levels of MmPrdx6 expression were higher than in the normal control from 24 to 38 h post-infection (Fig. 4b). In contrast to the S2-infected group, MmPrdx6 expression levels were upregulated in the *L. monocytogenes*-infected group from 2 to 6 h post-infection, followed by downregulation from 6 to 50 h post-infection and were higher than in the normal control from 4 to 6 h post-infection (Fig. 4c). The inactivated S2-infected group showed up-regulation of expression of MmPrdx6 beyond the level in the normal control (Fig. 4d). In general, the numbers of viable bacteria in RAW264.7 cells were decreased from 2 to 50 h post-infection in all groups. However, the numbers of viable bacteria in RAW264.7 macrophages were increased from 26 to 50 h post-infection in the S2-infected group and from 4 to 6 h post-infection in the *L. monocytogenes*-infected group (Fig. 4e).

### Bacterial survival in RAW264.7 cells accompanying overexpression of MmPrdx6

**Fig. 5:**
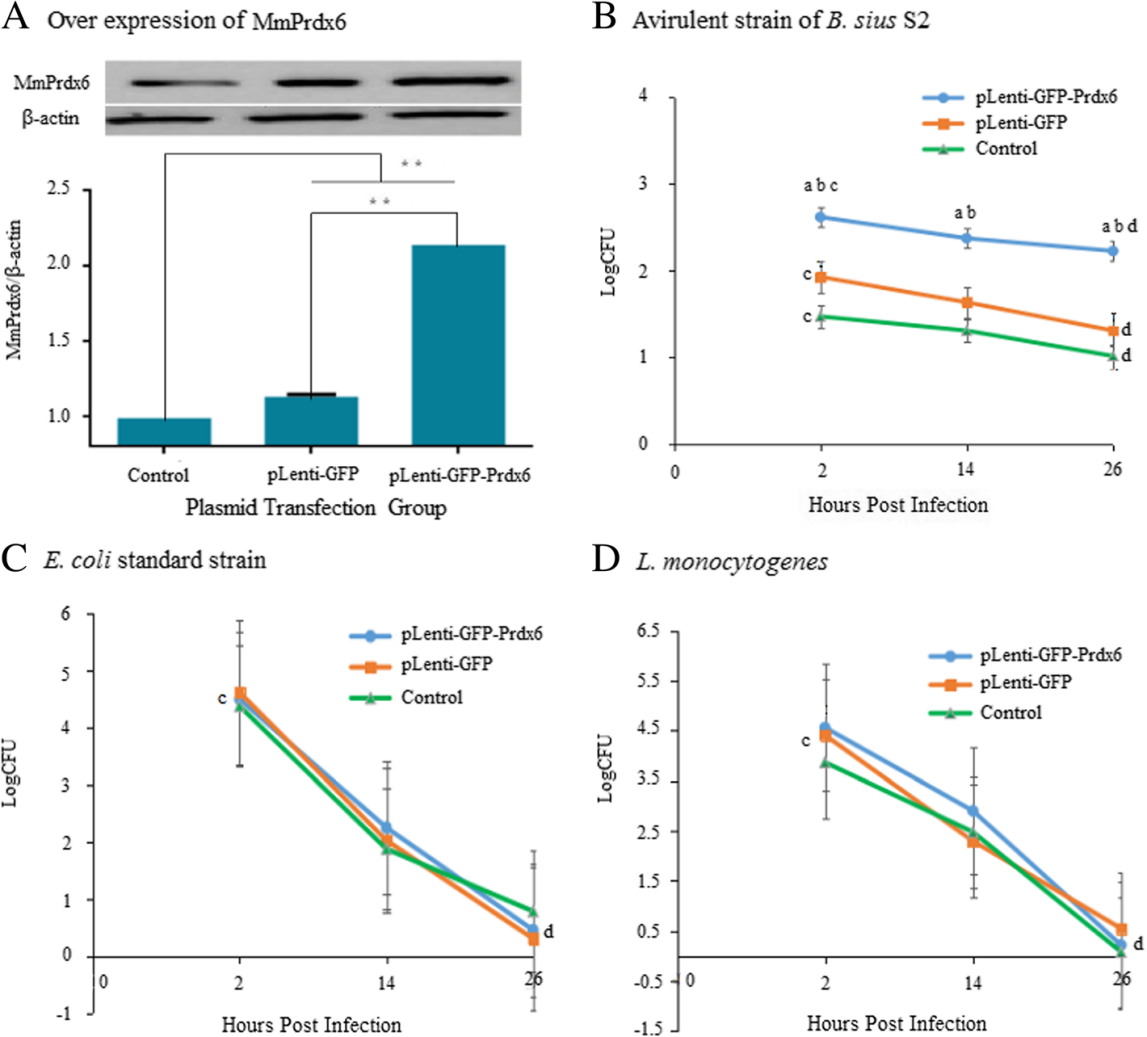
Overexpression of MmPrdx6 and counting of intracellular viable bacterial in RAW264.7 macrophages. **a** Levels of MmPrdx6 protein overexpression determined by western blotting. **b**-**d** Total number of intracellular viable bacteria in RAW264.7 macrophages overexpressing MmPrdx6 including the *B. suis* S2 strain (**b**), the *E. coli* standard strain (**c**) and *L. monocytogenes* (**d**). Statistical analysis was conducted by one-way analysis of variance (ANOVA) using SPSS 13.0 software. * *P* < 0.05, ** *P* < 0.01. a: *p* < 0.05 vs. the pLenti-GFP-transfected negative control; b: *p* < 0.05 vs. the untreated blank control; c: *p* < 0.05 vs. 26 h post infection in the same group.; d: *p* < 0.05 vs. 2 h post infection in the same group

The *MmPrdx6* ORF fragment was inserted into the pLenti-GIII-CMV-GFP-2A-puro plasmid, and the integrity of the recombinant plasmid for MmPrdx6 expression was verified via DNA sequencing, revealing 100% sequence identity to the ORF of the *MmPrdx6* gene (GenBank accession number AK168223.1), which demonstrated that the eukaryotic MmPrdx6 overexpression plasmid (*pLenti-GFP-Prdx6*) was successfully constructed. After transfection of the recombinant plasmids, MmPrdx6 overexpression was confirmed in RAW264.7 cells via western blotting (Fig. 5a).

The avirulent S2 strain of *B. suis*, *L. monocytogenes* and the *E. coli* standard strain were used to infect RAW264.7 macrophage cells transfected with MmPrdx6 overexpression plasmids, and the total counts of viable bacteria and Prdx6 expression levels were detected at 2, 14 and 26 h post-infection. The number of intracellular viable bacteria gradually decreased with extension of the cell culture time (Fig. 5b, c and d). However, the rate of the reduction of viable bacteria in the S2-infected group was lower than in the *L. monocytogenes*-infected and *E. coli*-infected groups, and the number of viable S2 in RAW264.7 cells was maintained at a relatively high level in comparison with *L. monocytogenes* and *E. coli* in the other two groups (Fig. 5). Additionally, in the S2-infected group (Fig. 5b), the number of viable *B. suis* S2 cells in RAW264.7 cells transfected with the MmPrdx6 overexpression vectors (plenti-GFP-Prdx6) was clearly higher than in the blank control (untreated RAW264.7 cells) and the negative control (RAW264.7 cells transfected with pLenti-GIII-CMV-GFP-2A-puro plasmids (plenti-GFP)). Nevertheless, in the other two *L. monocytogenes*- and *E. coli*-infected groups, transfection of the MmPrdx6 overexpression plasmids did not affect the count of viable bacteria in RAW264.7 cells compared to the blank control and the negative control (Fig. 5c and d). Therefore, the overexpression of MmPrdx6 contributed to the survival of the *B. suis* S2 strain in RAW264.7 cells.

### Bacterial survival in RAW264.7 cells accompanying knockdown of MmPrdx6

**Fig. 6:**
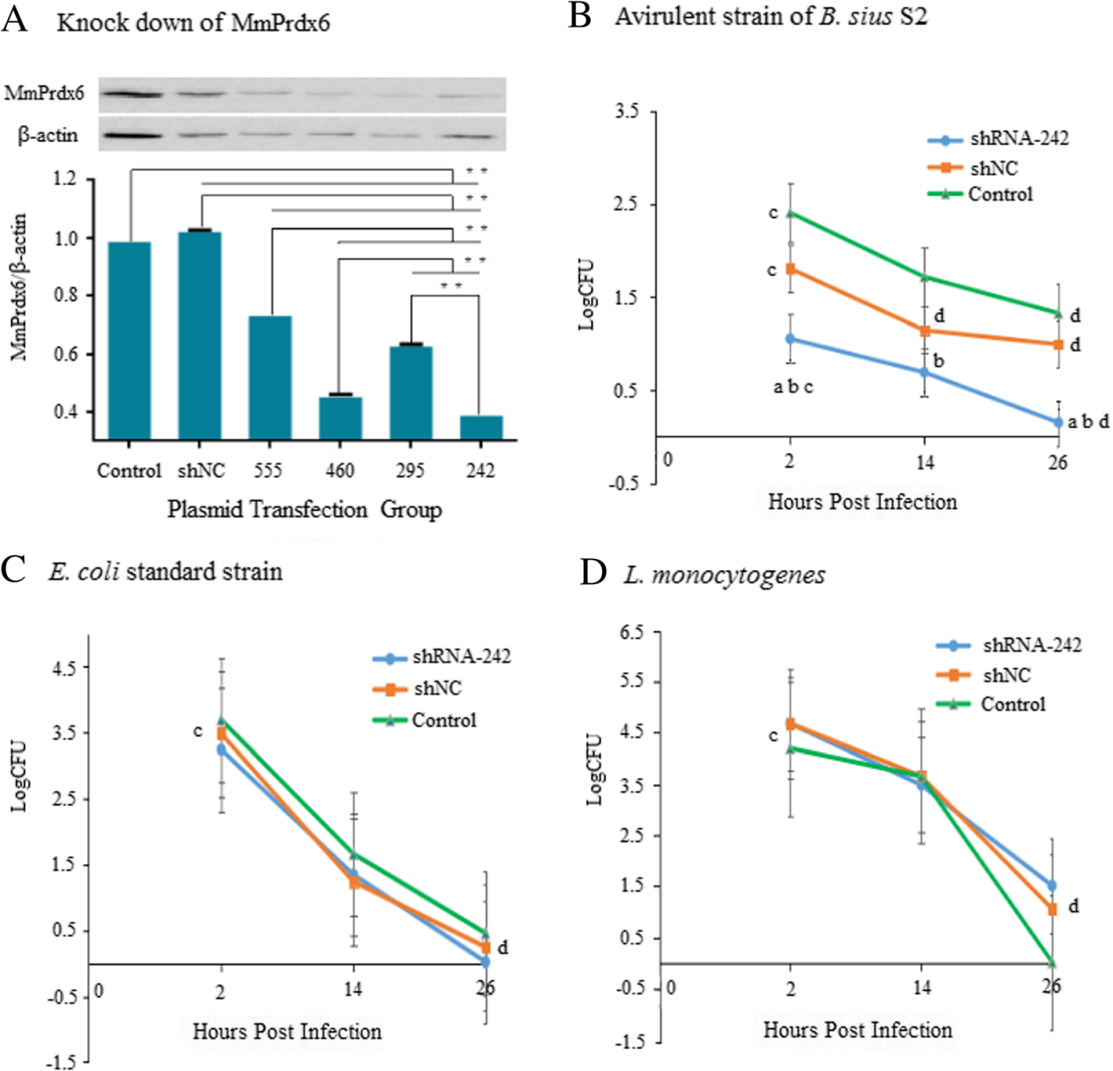
Knockdown of MmPrdx6 and counting of intracellular viable bacteria in RAW264.7 microphages. **a** Effective MmPrdx6-silencing plasmids analysed by western blotting. **b**-**d** Total number of intracellular viable bacteria in RAW264.7 macrophages in which MmPrdx6 expression was downregulated, including the *B. suis* S2 strain (**b**), the *E. coli* standard strain (**c**) and *L. monocytogenes* (**d**). Statistical analysis was conducted by one-way analysis of variance (ANOVA) using SPSS 13.0 software. * *P* < 0.05, ** *P* < 0.01. a: *p* < 0.05 vs. the shNC-transfected negative control; b: *p* < 0.05 vs. the untreated blank control; c: *p* < 0.05 vs. 26 h post infection in the same group; d: *p* < 0.05 vs. 2 h post infection in the same group

Four kinds of shRNA interference vectors and the pGPH1/GFP/Neo-shNC vector (shNC, negative control) were transfected into RAW264.7 cells; untreated RAW264.7 macrophage cells served as a blank control. Green fluorescence protein expressed from the transfected vectors in RAW264.7 cells was observed with a fluorescence microscope, which confirmed the successful transfection of shRNA interference vectors (data not shown). Through western blotting analysis, the expression of MmPrdx6 decreased in RAW264.7 cells after vector transfection in contrast to the blank control and the negative control, and Prdx6-Mus-242 shRNA transcribed from the pGPH1/GFP/Neo-Prdx6-Mus-242 vector (shRNA-242) was considered to be the most effective interfering shRNA (Fig. 6a).

The same three species of bacteria indicated above were used to infect the RAW264.7 murine macrophage cells transfected with shRNA-242. The total counts of viable bacteria and MmPrdx6 expression levels in RAW264.7 cells were detected at 2, 14 and 26 h post-infection. The counts of the three species of viable bacteria gradually decreased with extension of the cell culture time (Fig. 6b, c and d). However, intracellular viable *B. suis* S2 cells differed from the other two bacterial species in RAW264.7 macrophages. The count of viable bacteria in the *B. suis* S2-infected group was lower than in the other *L. monocytogenes*- and *E. coli*-infected groups (Fig. 6). In addition, in the S2-infected group (Fig. 6b), the count of the viable *B. suis* S2 strain in RAW264.7 cells subjected to Prdx6-Mus-242-mediated knockdown of MmPrdx6 was much lower than in the blank control (untreated RAW264.7 cells) and the negative control (RAW264.7 cells transfected with shNC). However, in the *L. monocytogenes*- and *E. coli*-infected groups, Prdx6-Mus-242-mediated knockdown of MmPrdx6 did not lead to a difference in the intracellular bacterial count, in contrast to the blank control and the negative control (Fig. 6c and d). As a result, knockdown of MmPrdx6 reduced the survival of the *B. suis* S2 strain in RAW264.7 cells.

## Discussion

Although brucellosis is a worldwide zoonotic infectious disease, little is known regarding the factors involved in the virulence of *Brucella*. Therefore, research on *Brucella* virulence-related proteins is of great significance. *Brucella* can invade the body through the mucosa or epithelium of the digestive tract and undergo transport to macrophage cells via the lymphatic system, after which *Brucella* parasites reproduce in macrophage cells through a special immune evasion mechanism [16]. In this study, we focused on the MmPrdx6 gene, which we screened in a previous study from a time-course SSH cDNA library of buffy coats from sheep infected with different virulent *Brucella* strains. *Brucella* mainly parasitize macrophage cells. Therefore, we selected the RAW264.7 murine macrophage cell line as a host to study the intracellular survival of the *B. suis* S2 strain in response to Prdx6. *L. monocytogenes* was used as an intracellular pathogen control, and the *E. coli* standard strain was used as a non-intracellular bacterial control, to analyse the specificity of the intracellular interaction between *B. suis* S2 strain and the expression levels of Prdx6.

A *Prdx6* gene ORF from *Mus musculus* was cloned, and the recombinant MmPrdx6 protein was produced, after which the DNA protection activity of rMmPrdx6 against metal-catalysed oxidation was verified. A monoclonal antibody against MmPrdx6 was purified, and its specificity was confirmed. Then, differential expression analysis of Prdx6 in macrophage cells was performed after infection with vaccine strain S2 of *B. suis*, *L. monocytogenes* and *E. coli*, and the effects of differential Prdx6 expression on the intracellular survival of bacteria were analysed to better understand the relationship between host Prdx6 and *Brucella* infection.

To prepare the monoclonal antibody against MmPrdx6, resoluble, high-purity bioactive MmPrdx6 had to be obtained. Therefore, a His-tag was added to the rMmPrdx6 protein to facilitate purification and enhance dissolvability. The higher expression of the rMmPrdx6 protein observed in *E. coli* BL21(DE3) cells could imply that *E. coli* BL21(DE3) cells were suitable as a host for the expression of MmPrdx6. It has been reported that Prdx6 proteins from various sources display DNA protection activity [17]. The DNA protection activity of rMmPrdx6 was verified in this study (Fig. 2d), showing that rMmPrdx6 could protect plasmid DNAs.

The spatial structure of rMmPrdx6 expressed by a prokaryotic expression system, in the absence of subsequent protein modifications and folding, may be not exactly the same as that of the native Prdx6 protein [18]. Moreover, Prdx6 mainly exhibits GSH peroxidase activity and phospholipase A2 activity in cells and is barely detectable in extracellular fluid under normal physiological conditions. Therefore, Prdx6 rarely has access to the humoural immune system [18]. Hence, bioactive soluble rMmPrdx6 may be an effective immunogen for preparing mAbs against native MmPrdx6.

Prdx6 is associated with host responses against the invasion of bacteria and viruses [19] and may be a candidate molecular biomarker and treatment target for some diseases [20]. However, the role of MmPrdx6 as a biomarker will require further study for validation. We analysed the effect of infection with the *B. suis* S2 strain on the expression of MmPrdx6 in the RAW264.7 murine macrophage cell line, compared with infection with the intracellular pathogen *L. monocytogenes*, the non-intracellular opportunistic pathogen *E. coli* and inactivated S2 (Fig. 3). The expression pattern of MmPrdx6 in the S2-infected group clearly differed from that in the other groups, showing that MmPrdx6 was first downregulated and then upregulated by S2; however, MmPrdx6 expression in S2-infected RAW264.7 cells was lower than in normal RAW264.7 cells at all time points. The differential expression of MmPrdx6 induced by S2 in RAW264.7 cells was consistent with our previous study showing that the transcription of OaPrdx6 in the buffy coats of sheep infected with S2 is downregulated in comparison with normal sheep [6]. However, the expression of MmPrdx6 induced by the infection of RAW264.7 cells with *L. monocytogenes* differed from that induced by S2 (despite the fact that *L. monocytogenes* is also an intracellular pathogen), but was similar to that induced by *E. coli*, showing gradual upregulation from expression levels below those in untreated RAW264.7 cells to an expression peak above that in normal RAW264.7 cells, followed by downregulation to a level below that in the normal control (Fig. 4b and c). The different pathogenic mechanisms between *Brucella* an*d L. monocytogenes*, which are both intracellular pathogens, might result in differential expression patterns of MmPrdx6. Furthermore, the MmPrdx6 expression pattern induced by S2 seemed to be specific, being characterized by levels below those in the normal control (Fig. 4a and c). Inactivated S2 induced higher expression of MmPrDx6 in infected RAW264.7 cells than in untreated cells, and intracellular Prdx6 levels also showed a clear upward trend and differed greatly from those in liver bacteria-infected RAW264.7 cells (Fig. 4d). One potential mechanism is that live S2 may suppress the expression of MmPrdx6 due to some unknown mechanism, while the inactivated organism is unable to do so. Although heat-inactivated S2 exhibits no vital signs, it retains LPS, the major component of the Gram-negative bacterial cell wall, which can trigger and accelerate the inflammatory cascade [21]. Reactive oxygen species (ROS) represent an important innate immune response to LPS [22], and MmPrdx6 with NSGPx activity may be upregulated to clear intracellular ROS to avoid damage to cells caused by a high concentration of ROS. This mechanism may explain how the host plays a major role in combating the invasion of inactive foreign substances to a certain extent. The failure of the attenuated *B. suis* S2 strain to promote MmPrdx6 expression may be the reason underlying the resultant phenotype. However, it was interesting that upregulation of MmPrdx6 occurred along with the increase in the liver bacterial count in intracellular liver pathogen-infected RAW264.7 cells, as observed in the S2-infected group from 38 to 50 h post-infection (Fig. 4a and e) and the *L. monocytogenes*-infected group from 4 to 6 h post-infection (Fig. 4c and e). For this reason, Prdx6 was inferred to contribute to the survival of intracellular pathogens, possibly because the intracellular pathogenic bacteria were not thoroughly killed by ROS, and a few surviving cells proliferated when the upregulated MmPrdx6 protein cleared ROS from cells. The MmPrdx6 expression patterns observed in the *L. monocytogenes-* and *E. coli*-infected groups suggested that liver *L. monocytogenes* and *E. coli* were phagocytized by macrophage cells to cause a brief burst of high ROS production to kill bacteria. At this early stage of infection, MmPrdx6 was not upregulated, thus this maybe allow sufficient ROS to exist in the cells to kill pathogens. After bacteria were eliminated, MmPrdx6 was upregulated above normal levels to clear ROS and peroxides. Thus, MmPrdx6 taking part in the host immune response against *L. monocytogenes* and *E. coli* did not show special functions, in contrast to other antioxidant enzymes such as peroxidases, SOD and CAT. However, the differential expression of MmPrdx6 induced by *Brucella spp.* seemed to be distinctive. Prdx6 expression levels and the count of viable bacteria in macrophage cells increased 26 h later in the S2-infected group (Fig. 4a and e), but these phenomena did not occur after infection with *L. monocytogenes* and *E. coli* (Fig. 4b, c and e). Additionally, it was interesting that the numbers of viable bacteria in RAW264.7 macrophages were increased from 26 to 50 h post-infection in the S2-infected group and from 4 to 6 h post-infection in the *L. monocytogenes*-infected group (Fig. 4e), accompanying the increase of MmPrdx6 expression. These results indicated that intracellular MmPrdx6 might be conducive to intracellular pathogen survival and proliferation in certain intracellular stages. As our previous study shown [6], the virulent *B. melitensis* can upregulate Prdx6 expression above the normal level, whereas the avirulent *B. suis* S2 strain downregulates its expression below the normal level. In this study, enhancement of intracellular Prdx6 expression can increase the number of S2 cells in macrophage cells. As a result, the expression of Prdx6 was important for intracellular survival of *B. suis* S2 strain and may be one of the reasons that virulent *Brucella* shows long-term survival in cells, while avirulent *Brucella* does not. Furthermore, it is possible that the expression of Prdx6 in host cells may be differentially regulated by different virulent *Brucella*, especially for the fully virulent *Brucella* to upregulate Prdx6 [6] for facilitating its own intracellular parasitism after the successful survival of a small number of *Brucella* in cells, but these assumptions must be verified through further study. Additionally, different expression patterns of OaPrdx6 in sheep buffy coats are observed in response to infection with different virulent strains of *Brucella* [6]. Therefore, investigation of the expression of intracellular Prdx6 as a means of evaluating the virulence of *Brucella* deserves further attention.

Nevertheless, it was indicated by the MmPrdx6 overexpression and knockdown experiments that intracellular MmPrdx6 might be conductive to *B. suis* S2 strain survival in cells. Independent of the overexpression and knockdown of Prdx6 (Figs. 5 and 6), the numbers of surviving *L. monocytogenes* and *E. coli* in macrophage cells were not related to the expression levels of intracellular Prdx6, as the counts of viable bacteria in all RAW264.7 cells examined (including those subjected to the recombinant plasmid-transfection test, the intact plasmid-transfected negative control, and the untreated blank control) were almost the same, without any significant difference. However, both the overexpression and knockdown of intracellular Prdx6 had an effect on the viable *B. suis* S2 strain. In macrophage cells with MmPrdx6 overexpression (Fig. 5b), the count of intracellular viable *B. suis* S2 strain was greater than in the blank control and the negative control, and the rate of decrease was low in comparison with *L. monocytogenes* and *E. coli*; at 26 h post-infection in particular, the count of viable S2 was greater than those of viable *E. coli* (Fig. 5c) and *L. monocytogenes* (Fig. 5d). Conversely, in macrophage cells subjected to MmPrdx6 knockdown (Fig. 6b), the number of intracellular viable *B. suis* S2 strain was lower than in the other two controls and was below the count of viable bacteria in the *E. coli*-infected (Fig. 6c) and *L. monocytogenes*-infected (Fig. 6d) groups. All of the above results showed that host Prdx6 contributed to the intracellular survival of *B. suis* S2 strain in infected cells. Hence, S2 might not only protect itself through host Prdx6 to achieve intracellular parasitism but may also utilize certain mechanisms to regulate the expression of host Prdx6 to strengthen this protective effect. However, the mechanism whereby Prdx6 contributes to the intracellular parasitism of *Brucella* remains to be further studied. Elucidation of the relationship between host Prdx6 and *Brucella* and the mechanism involved may facilitate the prevention and treatment of brucellosis in the future.

## Conclusions

In this study, recombinant mouse Prdx6 with antioxidant activity was expressed, and an anti- Prdx6 monoclonal antibody that bound native MmPrdx6 was prepared. Through infecting RAW264.7 macrophage cells with different bacteria (including the *B. suis* S2 strain, *L. monocytogenes* and *E. coli*) followed by detection of the differential expression of Prdx6, and through regulating the overexpression and knockdown of MmPrdx6 in macrophage cells followed by quantification of viable intracellular bacteria, it was confirmed that infection of cells with *L. monocytogenes* and *E. coli* showed little involvement of intracellular Prdx6, but host Prdx6 contributed to the intracellular survival of *B. suis* S2 strain in cells.

## Methods

### Reagents

Female BALB/c mice (LiaoNing ChangSheng Biotechnology Co., Ltd. China) were fed in cages under natural daylight with food and water available at all times and with an ambient temperature of 20 ± 2 °C and relative humidity of 50 ± 5% [23]. All of the animal experiments were carried out abiding by the provisions of the Institutional Animal Care and Use Committee of Jilin University (Nos. SCXK2015–0004 and 20,170,333). At the end of the study, all the animals were euthanized. The avirulent S2 strain of *Brucella suis* was purchased from Harbin Pharmaceutical Group Bioengineering Co., Ltd. (Haerbin, China). *Listeria monocytogenes* (CMCC540022), the *Escherichia coli* standard strain (CMCC44817), *Escherichia coli* DH5α (used for gene cloning), *E. coli* BL21(DE3); used for recombinant protein expression), the murine myeloma cell line SP2/0, the RAW264.7 murine macrophage cell line, and pLenti-GIII-CMV-GFP-2A-puro plasmids (abm, USA) were stored at the Institute of Zoonosis, Jilin University (China).

### Expression and bioactivity analysis of rMmPrdx6

A pair of primers (Mmprdx6s-up/Mnprdx6x-down) that introduced *Nde* I and *Hind* III restriction sites were designed based on the *MmPrdx6* gene (GenBank accession number AK168223.1) and synthesized by Sangon Biotech (Shanghai) Co., Ltd., China (Table 1). According to the manufacturer’s instructions, total RNAs from the RAW264.7 murine macrophage cell line were extracted using the TRIzol reagent (Invitrogen, USA) and reverse transcribed into cDNAs to be used as the PCR template for cloning the ORF of *MmPrdx6* with the PrimeScript™ 1st Strand cDNA Synthesis Kit (TaKaRa, Japan). PCR amplification was performed using the following protocol [24]. Briefly, each 50 μL reaction volume contained cDNA as the template (400 ng), 1.5 μL of 10 μM primers (Mmprdx6s-up / Mmprdx6x-down), 1.5 μL 2.5 mM dNTPs, 1 μL of HiFi hotstart (KAPA Biosystems), 10 μL of HiFi hotstart Fid buffer and 32.5 μL of water. The reaction conditions were as follows: 94 °C for 3 min; 32 cycles of 30 s at 94 °C, 30 s at 68 °C, and 50 s at 72 °C; then a final extension at 72 °C for 3 min. The PCR products were analysed via 1.0% agarose gel electrophoresis and purified with a QIA quick DNA gel extraction kit (Qiagen, Germany). After purification and ligation into the pMD18-T vector (TaKaRa, Dalian, China), the target DNA products were transformed into *E. coli* DH5α competent cells and sequenced by Shanghai Sangon Biological Engineering Technology & Service Co., Ltd. (Shanghai, China). The recombinant plasmids were then subjected to double digestion with the *Nde* I and *Hind* III restriction enzymes and subcloned into the pET-28a(+) expression vector (Novagen, USA) via standard molecular biology techniques [25]. Positive colonies carrying *pET-28a-Prdx6* expression plasmids with the *MmPrdx6* ORF insert were sequenced.

**Table 1:** Primers used in the present study

Primers	Sequences (5′–3′)
Mmprdx6s	CGCCATATGATGCCCGGAGGGTTGCTTCT
Mmprdx6x	CCCAAGCTTTTAAGGCTGGGGTGTATAACGGAGGTA
ZMprdx6s	CCGGATATCATGCCCGGAGGGTTGCTTCT
ZMprdx6x	TGCTCTAGATTAAGGCTGGGGTGTATAACGGAGGTA

The *pET-28a-Prdx6* recombinant expression plasmids were transformed into *E. coli* BL21(DE3). A single transformant colony was then cultured overnight in 5 mL LB medium containing 50 μg/mL kanamycin at 37 °C, and MmPrdx6 protein expression was induced by adding IPTG at a final concentration of 1 mM, at 37 °C and 180 rpm for 6 h. The bacteria were then collected by centrifugation (12,000 g for 1 min at 4 °C), after which the bacterial pellets were dissolved in 10 mL of lysis buffer (20 mM NaPO4 (pH 7.5) and 500 mM NaCl), and a 10 μL aliquot of the bacterial suspension was analysed by 12% SDS-PAGE. The bacterial samples were lysed by sonication on ice for 10 min at 50% output (200 W; work, 5 s; and interval, 5 s), and the supernatant was harvested to purify the recombinant MmPrdx6 protein using His Trap FF affinity columns pre-charged with Ni2+ ions (GE, USA) according to the manufacturer’s instructions. Then, 12% SDS-PAGE was used to analyse the efficiency of rMmPrdx6 purification, and the purified MmPrdx6 proteins were dissolved in 0.01 M PBS and stored at − 80 °C.

A metal-catalysed oxidation (MCO) assay was conducted using supercoiled DNAs of pCMV-Script plasmids as reaction substrates to determine the DNA protection effect of MmPrdx6 according to a previous study [17]. The pCMV-Script plasmids were extracted and purified using an Axy Prep Plasmid Miniprep Kit (Axygen, USA) according to the manufacturer’s instructions. Thereafter, 50 μL aliquots of reaction mixtures containing MCO (3 M NaCl, 0.6 mM FeCl3 and 0.1 M DTT were dissolved in 7 mM phosphate buffer), pCMV-Script plasmids (300 ng) and different concentrations of purified rMmPrdx6 (0.08 g/mL, 0.16 g/mL and 0.32 g/mL) were incubated at 37 °C for 1 h. The DNA protection effect was assessed by running 0.8% agarose gel electrophoresis. PBS and bovine serum albumin (BSA) were assayed separately under the same reaction conditions as the blank control and negative control.

### Preparation and specific analysis of monoclonal antibodies against MmPrdx6

The Committee on Ethics and Experimentation of Jilin University approved all involved experimental use of animals. Mice were euthanized by a physical method of cervical dislocation under the condition of unconsciousness cauesd by inhaling diethyl ether (medical gauze stained with 20 mL) in a sealed container of 200 mL [26, 27]. Fifty microgram of rMmPrdx6 emulsified with an equal volume of complete Freund’s adjuvant (Sigma, USA) was injected into footpad of mature female BALB/c mice. One week later, a booster injection with the same dose of rMmPrdx6 in incomplete Freund’s adjuvant (Sigma, USA) wasl carried out. Finally, the blood and spleens of the immunized mice were collected 1 week since the last booster. In the presence of PEG1000, the isolated spleen cells were fused at a ratio of 10:1 with myeloma cells (SP2/0). Then, the treated cells were cultured in 1640 cell culture medium (HyClone) containing 20% foetal bovine serum (FBS) and selective HAT medium (Sigma, USA), which changed per 4 days. Fourteen days later, limiting dilution (at least three times) was used to screen hybridoma cells secreting the anti-MmPrdx6 mAb. and ELISA for testing the culture supernatant of the positive hybridoma cells. The anti-MmPrdx6 mAb was purified from the ascites of mice inoculated with positive hybridoma cells, and its subtype was identified with a mouse mAb Ig class/subclass ELISA kit (Ze Yu Biotechnology Co., Ltd. China) according to the manufacturer’s instructions. Native Prdx6 in the RAW264.7 murine macrophage cell line was tested by binding with the prepared anti-MmPrdx6 mAb via a routine western blotting procedure to analyse mAb specificity. The results were visualized by Enhanced Chemiluminescence Substrate Reagent (BeyoECL Plus, Beyotime, China) with an ECL Detection System (Microchemi 4.2, DNR, Israel).

### Differential expression of MmPrdx6 in RAW264.7 cells after infection with bacteria

To determine whether host Prdx6 has a specific effect on *B. suis* S2 strain, *L. monocytogenes* was selected as an intracellular virulent pathogen control [28], and *E. coli* was used as a non-intracellular bacterial control [29]. The avirulent *B. suis* S2 strain, *L. monocytogenes* and the *E. coli* standard strain were used to infect the RAW264.7 murine macrophage cell line. S2 in Trypticase Soy Broth (TSB) (Difco, USA), and *L. monocytogenes* and the *E. coli* standard strain were cultured in LB at 37 °C and 180 rpm to an appropriate concentration. Thereafter, a 5 mL aliquot of S2 was inactivated in a water bath at 80 °C for 10 min.

The RAW264.7 macrophages were resuscitated and cultured in 24-well cell culture plates to 2 × 10^5^ cells per well. Two hours before bacterial infection, DMEM (10% FBS) containing 100 units/ml penicillin-streptomycin (HyClone) was replaced with penicillin-streptomycin-free DMEM (10% FBS) medium, and RAW264.7 cells were then infected with bacteria at a ratio of 1:100 and incubated for 1 h in a cell culture incubator at 37 °C under 5% CO2. Next, the cells were washed twice with DMEM, after which DMEM containing 10% FBS and 150 units/ml penicillin-streptomycin was used to culture the cells. Infected RAW264.7 macrophages were collected at 2, 4, 6, 10, 14, 26, 38 and 50 h post-infection and lysed with 0.1% (V/V) Triton X-100 (Sigma–Aldrich) to detect the change in MmPrdx6 by western blotting, and the total number of viable intracellular bacteria was measured via the plate counting method. Six repetitions (three for western blotting and three for plate counting) were performed at each time point, and this assay was repeated three times. Image_j view analysis software was used to analyse the experimental results. Statistical analysis was carried out through one-way ANOVA using SPSS 13.0 software.

### Overexpression of MmPrdx6 in RAW264.7 cells and intracellular infection of bacteria

The MmPrdx6 overexpression vectors for RAW264.7 cells consisted of pLenti-GIII-CMV-GFP-2A-puro plasmids into which the *MmPrdx6* ORF was introduced along with *EcoR*V and *Xba*Irestriction sites via PCR with of the ZMprdx6s-up / ZMprdx6x-down primer pair (Table 1). The recombinant MmPrdx6 overexpression plasmid (pLenti-GFP-Prdx6) was confirmed to carry the correct ORF by sequencing at the Sangon Biotech (Shanghai) Co., Ltd., China. Endotoxin-free plasmids used for cell transfection were extracted with an EndoFree Plasmid Maxi Kit (Qiagen, German) according to the manufacturer’s instructions. The recombinant Prdx6 overexpression plasmids and pLenti-GIII-CMV-GFP-2A-puro plasmids (pLenti-GFP) (as the negative control) were transfected into RAW264.7 cells using the FuGENE HD transfection agent (Promega, USA), and the levels of overexpressed MmPrdx6 were detected by western blotting. Three species of bacteria, including the *B. suis* S2 strain, *L. monocytogenes* and the *E. coli* standard strain, were used to infect the MmPrdx6-overexpressing RAW264.7 macrophages, and the total numbers of intracellular viable bacteria at 2, 14 and 26 h post-infection were counted and analysed as described in the section on Differential expression of MmPrdx6 in RAW264.7 cells after infection with bacteria.

### Knockdown of MmPrdx6 in RAW264.7 cells and intracellular infection of bacteria

Four kinds of short hairpin RNA (shRNA) interference vectors were designed for knockdown of MmPrdx6 in RAW264.7 cells and synthesized by Shanghai GenePharma Co., Ltd., and the corresponding four interfering shRNA sequences are shown in Table 2. The pGPH1/GFP/Neo-shNC vector (shNC) was used as the negative control. Endotoxin-free shRNA interference plasmids were extracted using the EndoFree Plasmid Maxi Kit (Qiagen, German) and transfected into RAW264.7 cells to screen effective shRNA interference vectors for knockdown of MmPrdx6. Then, RAW264.7 macrophages transfected with the effective shRNA plasmids were infected with the avirulent S2 strain of *B. suis*, *L. monocytogenes* and the *E. coli* standard strain, after which the total numbers of intracellular viable bacteria at 2, 14 and 26 h post-infection were counted and analysed as described in section 2.4.

**Table 2:** Sequence information of the interference shRNAs

Names of shRNAs	Sequences (5′–3′)	Corresponding shRNA interference vectors
Prdx6-Mus-242	GAACTTGGCAGAGCTGCAAAG	pGPH1/GFP/Neo-Prdx6-Mus-242
Prdx6-Mus-295	GTTGATTGCTCTTTCAATAGA	pGPH1/GFP/Neo-Prdx6-Mus-295
Prdx6-Mus-460	GGACGCTAACAACATGCCTGT	pGPH1/GFP/Neo-Prdx6-Mus-460
Prdx6-Mus-555	GCAGGAACTTTGATGAGATTC	pGPH1/GFP/Neo-Prdx6-Mus-555

## Data Availability

The datasets used and/or analysed during the current study are available from the corresponding author upon reasonable request.

## Abbreviations

CAT: Catalase
cDNA: Complementary DNA
DMEM: Dulbecco’s modified eagle medium
ELISA: Enzyme linked immunosorbent assay
HAT: Hypoxanthine, aminopterin and thymidine
MmPrdx6: *Mus musculus* Prdx6
NSGPx: GSH peroxidase
OaPrdx6: *Ovis aries* Prdx6
PBS: Phosphate buffered solution
PLA2: Phospholipase A2
Prdx6: Peroxiredoxin 6
PVDF: Polyvinylidene fluoride
rMmPrdx6: Recombinant protein MmPrdx6
SDS: Sodium Dodecyl Sulfonate
shNC: pGPH1/GFP/Neo-shNC vector
shRNA-242: pGPH1/GFP/Neo-Prdx6-Mus-242 vector
